# Evolution of Neonatal Imitation

**DOI:** 10.1371/journal.pbio.0040311

**Published:** 2006-09-05

**Authors:** Liza Gross

Humans do it. Chimps do it. Why shouldn’t monkeys do it, too? Mimicry exists throughout the animal kingdom, but imitation with a purpose—matching one’s behavior to others’ as a form of social learning—has been seen only in great apes. (Mockingbirds can imitate an impressive number of other birds’ songs, but they can’t mimic you sticking out your tongue like a chimp can.) This matching behavior likely helps individuals conform to social norms and perform actions in the proper context. It’s generally believed that monkeys do not imitate in this way. However, the discovery that rhesus monkeys have “mirror neurons”—neurons that fire both when monkeys watch another animal perform an action and when they perform the same action—suggests they possess the common neural framework for perception and action that is associated with imitation.

**Figure:**
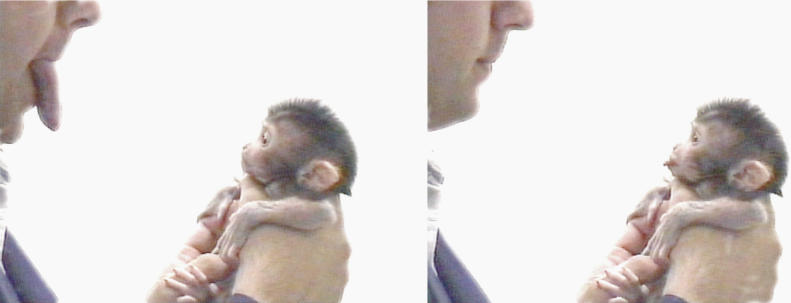
A newborn macaque imitates tongue protrusion

Most studies exploring the early signs of matching behavior have focused on humans. A landmark 1977 study by Andrew Meltzoff and Keith Moore showed that 12- to 21-day-old infants could imitate adults who pursed their lips, stuck out their tongue, opened their mouth, and extended their fingers. They later found similar results in newborns, demonstrating that imitation is innate, not learned. A handful of studies on newborn chimps found a similar capacity for imitating human facial gestures. In a new study, Pier Ferrari, Stephen Suomi, and colleagues explored the possibility that imitation evolved earlier in the primate tree by studying neonatal imitation in rhesus monkeys, which split from the human lineage about 25 million years ago. They found that rhesus infants can indeed imitate a subset of human facial gestures—gestures the monkeys use to communicate. The first investigation of neonatal imitation outside the great ape lineage, their study suggests that the trait is not unique to great apes after all.

Ferrari et al. tested 21 baby rhesus monkeys’ response to various experimental conditions at different ages (one, three, seven, and 14 days old). Infants were held in front of a researcher who began with a passive expression (the baseline condition) and then made one of several gestures, including tongue protrusion, mouth opening, lip smacking, and hand opening.

Day-old infants rarely displayed mouth opening behavior, but smacked their lips frequently. When experimenters performed the mouth opening gesture, infants responded with increased lip smacking but did not increase any other behavior. None of the other stimuli produced significant responses. But by day 3, matched behaviors emerged: infants stuck out their tongues far more often in response to researchers’ tongue protrusions compared with control conditions, and smacked their lips far more often while watching researchers smacking theirs. (Watch an infant imitating mouth opening at DOI: 10.1371/journal.pbio.0040302.sv001.) By day 7, the monkeys tended to decrease lip smacking when humans performed the gesture, and by two weeks, all imitative behavior stopped.

Infant rhesus monkeys, these results suggest, have a narrow imitation window that opens three days after birth, when they can reproduce human tongue protrusion and lip smacking. This imitation period is much longer in humans (two to three months) and chimps (about two months). It’s possible that rhesus babies show more varied and prolonged imitative behavior in response to mom or other monkeys than to human experimenters, who may not provide the most relevant biological cues. But this narrow window does comport with the development schedule of rhesus monkeys, which is much shorter than that of humans and chimps.

Many questions remain about the neural mechanisms of neonatal imitation. The researchers argue that their results support a resonance mechanism linked to mirror neurons, which have recently been identified while monkeys observe others’ lip smacking and tongue protrusion. In this model, observing human mouth gestures directly activates mirror neurons in the monkeys’ brain, ultimately leading to a replication of the gesture.

Human babies can imitate an adult’s facial gesture a day after seeing it, which may help them identify individuals. For rhesus monkeys, lip smacking (which often alternates with tongue protrusion) accompanies grooming sessions and signals affiliation—an important social cue for a species that is often described as “despotic and nepotistic.” Picking up these social gestures early in life may well facilitate the animal’s early social relations (primarily with the mother) and assimilation into the social fabric of the group, providing a mechanism for distinguishing friend from foe. It will be interesting to test the extent of imitation in monkeys with more complex social dynamics. While the social life of rhesus monkeys may not demand the more sophisticated repertoire of behaviors seen in great apes, they seem to be hard-wired for imitation just like apes.

